# Supplementary material for: Funders’ data-sharing policies in therapeutic research: A survey of commercial and non-commercial funders
Source: PLoS One. 2020 Aug 20;15(8):e0237464. doi: 10.1371/journal.pone.0237464 (PMC7446799; doi:10.1371/journal.pone.0237464)
Supplement: Attachment — Submitted filename: responsestoreviewers_plosOne.docx [file pone.0237464.s002.docx]

Words in blue: Responses to the reviewers’ comments

Words in green: Text with corrections in line with the comments of the reviewers

Words highlighted in yellow: Modifications in line with the comments of the reviewers

*Text in italics: Reviewers' comments*

Dear Editor,

Thank you for giving us the opportunity to submit a revised manuscript. We really appreciate the time that you and the reviewers have devoted to providing valuable feedback on our manuscript. Please find here responses to the reviewer ‘comments.

*Reviewer #1 :*

*This research aimed to describe commercial and non-commercial funders' data-sharing policies and to assess the compliance of funded RCTs with the existing data-sharing policies. I feel this article satisfies the PLOS ONE criteria (see below)

1. The study presents the results of original research. Yes
2. Results reported have not been published elsewhere. Yes
3. Experiments, statistics, and other analyses are performed to a high technical standard and are described in sufficient detail. Yes
4. Conclusions are presented in an appropriate fashion and are supported by the data. Yes
5. The article is presented in an intelligible fashion and is written in standard English. Yes
6. The research meets all applicable standards for the ethics of experimentation and research integrity. Yes
7. The article adheres to appropriate reporting guidelines and community standards for data availability. N/A

Overall, the methodology of this study is robust and appears to be consistent with a previous study published by this research team (Data sharing and reanalysis of randomized controlled trials in leading biomedical journals with a full data sharing policy: survey of studies published in The BMJ and PLOS Medicine). The article is well-written and concise. My only suggestion would be the authors consider amending the title of this study from audit to survey. This article is not really an audit as the results are not compared against an evidence based standard.*

We are very grateful for your encouraging comments and valuable feedback on our work. We are delighted to hear that you find our work well-written and concise.

Thank you for this suggestion to amending the title of the article. We agree with you. The word “audit” has been replaced by the word “survey” in the title and in the main text.

Funders' data-sharing policies in therapeutic research: a survey of commercial and non-commercial funders

“As our survey points out, funders do not provide for incentives for data-sharing, and funding specially dedicated to data-sharing in not put in place by all of them. This lack of incentives and funding could hinder the implementation of the policies put in place. It is also possible that trialists registering their trial on clinicaltrials.gov do not attach importance to data-sharing plans at the time of registration.”
